# Alterations in functional and structural connectivity in the 6-OHDA-induced Parkinsonian rat model

**DOI:** 10.3389/fnins.2025.1591215

**Published:** 2025-06-03

**Authors:** Shuyi Zhu, Maurizio Bergamino, Alberto Fuentes, Ivette M. Sandoval, David J. Marmion, Christopher Bishop, Fredric P. Manfredsson, Ashley M. Stokes

**Affiliations:** ^1^Barrow Neuroimaging Innovation Center, Barrow Neurological Institute, Phoenix, AZ, United States; ^2^School of Life Sciences, Arizona State University, Tempe, AZ, United States; ^3^Department of Psychology, Binghamton University, Binghamton, NY, United States

**Keywords:** Parkinson's disease, 6-hydroxydopamine, functional connectivity, structural connectivity, functional magnetic resonance imaging, diffusion magnetic resonance imaging, free water diffusion tensor imaging

## Abstract

**Introduction:**

Parkinson's Disease (PD), the second most common neurodegenerative disorder, is characterized by motor and non-motor symptoms linked to dopaminergic neuron degeneration. This study utilized the 6-hydroxydopamine (6-OHDA) rat model to replicate PD-like dopaminergic degeneration through targeted injections into the medial forebrain bundle and substantia nigra.

**Methods:**

Behavioral assessments revealed hallmark motor deficits, while MRI was performed to assess complementary functional connectivity and structural connectivity. Post-mortem tyrosine hydroxylase (TH) staining confirmed extensive dopaminergic neuron loss, validating the pathological relevance of the model and ensuring data integrity. MRI data were collected at 7T in 46 male Fischer F344 rats (23 6-OHDA, 23 sham) to characterize functional and structural connectivity differences between cohorts.

**Results:**

Functionally, decreased connectivity between the retrosplenial and endopiriform cortices in the 6-OHDA model suggests disrupted sensory processing, while increased connectivity between the hippocampus and retrosplenial cortex indicates possible compensatory mechanisms. Structurally, we observed reduced connectivity between the subcoeruleum and piriform cortex in the 6-OHDA model, which may reflect axonal degeneration, and increased connectivity between the ventral striatum and primary somatosensory cortex, which likely reflects compensatory changes to support motor-sensory integration. Diffusion MRI analysis further revealed changes in the white matter tracts connecting these regions, supporting these findings and highlighting adaptive responses to neurodegeneration in PD.

**Discussion:**

These findings demonstrate the utility of combining functional and structural connectivity analyses to capture PD-related network disruptions. These structural connectivity changes were further associated with microstructural alterations. The development of MRI biomarkers for understanding brain connectivity may enhance our understanding of PD pathology and advancing translation of these techniques to clinical applications.

## 1 Introduction

Parkinson's Disease (PD) is the second most common neurodegenerative disorder worldwide, affecting ~1% of the population over 60 years, with increasing prevalence of PD with age (Tysnes and Storstein, 2017). The primary neuropathological feature is the loss of dopaminergic neurons in the substantia nigra (SN) and the aggregation of misfolded alpha-synuclein proteins (Zhou et al., 2023; Ganguly et al., 2021). These changes result in the classic motor symptoms of bradykinesia, tremor, and rigidity, as well as non-motor symptoms such as autonomic dysfunction, cognitive impairment, and mood disorders (Postuma et al., 2015). The loss of dopaminergic neurons, leading to dopamine deficiency, is regarded as the primary driver of motor symptoms (Zhou et al., 2023). Additionally, non-motor symptoms have been linked to patterns of dopamine depletion, particularly in the associative and limbic striatum, although non-dopaminergic mechanisms may also contribute to these symptoms (Chung et al., 2020). Alpha-synuclein pathology, as another critical factor, is associated with both motor and non-motor symptoms, further emphasizing the interplay of multiple pathological processes (Ganguly et al., 2021).

Non-invasive neuroimaging methods, such as magnetic resonance imaging (MRI), play a crucial role in exploring both structural and functional alterations in PD (Prange et al., 2019; Chen et al., 2022). MRI can be a vital tool for quantifying early changes in PD due to its high spatial resolution and non-invasiveness. By optimizing imaging parameters and exploiting various contrasts, MRI offers the flexibility to generate multiple forms of contrast that can be utilized to characterize pathological changes; such MRI methods have enabled detection of subtle pathological changes including decreased gray matter volume, increased iron content in the SN, and disrupted brain connectivity patterns (Ganguly et al., 2021; Prange et al., 2019; Chen et al., 2022).

Functional MRI (fMRI) measures and maps brain activity by detecting changes in blood flow associated with neuronal activation, thus offering insights into the functional alterations in PD (Buxton, 2013). Resting-state fMRI (rs-fMRI) correlates the signals from distinct brain areas to understand how these regions interact and form networks (Buxton, 2013). In the symptomatic stages of PD, atypical functional connectivity (FC) has been detected in various PD-related networks, including the default mode network (DMN), executive control network (ECN), salience network (SalN), and sensorimotor network (SMN) (Zhang et al., 2021; Chen et al., 2022). However, results vary across different disease stages and patient subtypes (Filippi et al., 2021). For instance, decreased DMN connectivity has been associated with deficits in working memory and attention in non-demented PD patients, while increased DMN and ECN connectivity have been linked to motor severity (Smallwood et al., 2021; Chen et al., 2022). On the other hand, in the preclinical phase of PD, rs-fMRI can detect subtle changes in brain connectivity patterns, such as decreased functional connectivity in supplementary motor area, dorsolateral prefrontal cortex and putamen (Sang et al., 2015). Many of these early changes occur before overt motor symptoms manifest and are likely a compensatory reorganization of the brain functional architecture.

Diffusion MRI (dMRI) is sensitive to microscopic water movements within the brain, which can be used to infer white matter (WM) fiber connectivity (Bergamino et al., 2020). The dMRI-based technique diffusion tensor imaging (DTI) has been widely used to reconstruct and analyze the structural connectivity (SC) in PD and other neurodegenerative disorders (Yang et al., 2021; Bergamino et al., 2023). Previous research in PD shows widespread decreased structural connectivity, including the cortico-striatal pathway, frontoparietal-striatal pathway, and nigro-pallidal pathway (Barbagallo et al., 2017; Tinaz et al., 2017; Yang et al., 2021; Bergamino et al., 2023). Other studies have demonstrated reduced structural connectivity associated with limbic and mesolimbic pathways, which can be functionally and anatomically connected to the SN and SN-associated regions (Bergamino et al., 2023).

Although previous studies using dMRI have identified significant differences in PD patients, these findings are not yet sufficient to reliably diagnose the disease (Deng et al., 2018; Arribarat et al., 2019; Yang et al., 2021). This limitation may be attributed to methodological challenges such as the impact of partial volume effects (PVEs), which reduce the specificity of DTI metrics to individual tissue types and thus reduce the accuracy of the resulting metrics (Bergamino et al., 2021; Novikov, 2021). To overcome this limitation, free-water (fw) correction algorithms for DTI (fw-DTI) have been developed (Pasternak et al., 2009). These algorithms yield both fw-corrected metrics and the fw index (f), where the latter is expected to increase with neuroinflammation and atrophy-based neurodegeneration (Pasternak et al., 2009). In PD patients, studies show that f is elevated in specific brain regions such as the SN, globus pallidus and cerebellar lobule, further correlating with dopaminergic deficiency and disease progression (Ofori et al., 2015b; Planetta et al., 2016; Arribarat et al., 2019; Zhou et al., 2021). While fw-DTI can improve specificity compared to conventional DTI, it is important to note that most clinical and preclinical applications, including this study, often rely on single-shell dMRI acquisitions. Although single-shell fw-DTI remains widely used and provides valuable insights, it offers somewhat lower accuracy in separating free-water and tissue compartments compared to multi-shell approaches (Pasternak et al., 2012). Therefore, findings derived from single-shell fw-DTI should be interpreted with appropriate caution.

Preclinical animal models, such as the 6-hydroxydopamine (6-OHDA) model, are often used to explore PD pathophysiology. This model effectively replicates the degeneration of the nigrostriatal dopamine system and demonstrates robust functional brain networks that are analogous to those seen in human disease (Sierakowiak et al., 2015; Perlbarg et al., 2018). Previous investigations utilizing this model have revealed significant alterations in both DTI metrics and FC. For instance, Perlbarg et al. demonstrated that DTI biomarkers reliably indicated longitudinal neurodegeneration within the cortico-basal ganglia network; additionally, these biomarkers correlated with quantitative post-mortem histology (Perlbarg et al., 2018). Another recent study revealed free water increases in the subthalamic nucleus and brainstem (Bergamino et al., 2025). In the SN, microstructural alterations using dMRI have been observed, and these changes also correlated with motor impairments (Monnot et al., 2017; Zhang et al., 2017). Furthermore, decreased FC is observed in cortico-cortical and striato-cortical connections, specifically between the ipsilateral primary motor cortex (M1) and contralateral thalamus (TH), in the interhemispheric striatum (STR), and within the ipsilateral cortices of 6-OHDA rats, which is commonly interpreted as direct lesioning effects (Monnot et al., 2017; Perlbarg et al., 2018; Zhurakovskaya et al., 2019; Petiet, 2021). Studies show that SC and FC exhibit a robust correlation in healthy rats, indicating a mutual interaction between anatomical networks and functional activity in rats (Díaz-Parra et al., 2017; Straathof et al., 2020). Therefore, it is hypothesized that such a relationship might persist in pathological situations like PD. Combining both approaches could provide valuable insights in understanding PD progression.

In this study, we used the 6-OHDA lesion model to compare the SC and FC profiles between lesioned rats and sham-operated controls. We identified fiber bundles exhibiting significant connectivity discrepancies, for which we computed the f-index to quantitatively assess the degree of microstructural impairment. This dual-modality approach highlights the bidirectional influence between structural and functional brain networks and allows for a detailed examination of the disruption caused by PD. Our findings may contribute to a broader understanding of how structural degeneration in PD is mirrored in functional alterations, including compensatory mechanisms that emerge to mitigate the effects of neural loss, thus enhancing our knowledge of disease progression mechanisms and facilitating the identification of potential biomarkers and therapeutic targets.

## 2 Methods

### 2.1 Animals

The Barrow Neurological Institute Institutional Animal Care and Use Committee (IACUC) approved the study (approval number: 555, approved on March 17, 2021). A total of 52 adult male Fischer 344 rats, weighing approximately between 280 and 320 g at the start of the study, were included. The rats were housed in pairs or trios in a temperature-controlled (22°C ± 1°C) and light-controlled room with a 12-h light/dark cycle. Food and water were available *ad libitum*.

During stereotaxic surgery under isoflurane anesthesia (2%), animals received two unilateral injections into the left medial forebrain bundle (MFB; Bregma −4.3 mm AP, +1.6 mm L, −8.4 mm DV from the skull) and substantia nigra (SN; Bregma −4.8 mm AP, +1.7 mm L, −8.0 mm DV from the skull). They were administered either 6-OHDA-hydrobromide (10 μg per injection, 5 mg/ml in 0.2 mg/ml ascorbic acid in 0.9% saline; *n* = 27) or vehicle (0.2 mg/ml ascorbic acid in 0.9% saline; *n* = 25) delivered using a glass capillary fitted on a 10 μl Hamilton Syringe (Benskey and Manfredsson, 2016). For each site, 2 μl was delivered at a rate of 0.5 μl/min (Benskey and Manfredsson, 2016; Sellnow et al., 2020); following the injection, the needle was left in place for 2 min to prevent backflow. Approximately 21 days later (21.03 ± 3.9 days), all rats underwent behavioral tests and MRI.

### 2.2 Behavioral tests

The success of nigrostriatal lesioning was confirmed using the cylinder test, which is often used to evaluate motor changes as a result of severe nigrostriatal denervation in Parkinsonian models (Steece-Collier et al., 2019). Spontaneous forelimb use was recorded until the rat performed a minimum of 20 weight-bearing forepaw contacts on the cylinder wall. The number of contacts made with the left, right, and both forelimbs on the cylinder wall was enumerated and data were tabulated as [(contralateral + ½ both) / (ipsilateral + contralateral + both)] × 100. A score of 50% is considered normal motor symmetry. Scores lower than 50% indicate reduced use of the contralateral forelimb, reflecting motor deficits in the limb opposite to the lesion site.

### 2.3 Data acquisition

MRI was conducted using a 7T Bruker Biospec USR 70/30 MRI (Billerica, MA) under isoflurane (3% for induction and 1.5% for maintenance, delivered in medical air, 1.5 L/min) using a Bruker 70 mm volume transmit and rat brain surface receive coil. A T1-weighted gradient-echo imaging with flow compensation (GEFC) sequence was performed to obtain three-dimensional (3D) axial images. The following imaging parameters were used: echo time (TE): 6 ms, repetition time (TR): 31.4 ms, spatial resolution of 0.2 × 0.2 × 0.4 mm^3^, flip angle: 15°, four averages, field of view (FOV) of 3 × 3 cm, and acquisition time of 13.5 min.

A multi-echo, multi-contrast EPI method was used to capture fMRI data, consisting of two echoes following an excitation pulse (gradient-echo) and two echoes following a refocusing pulse (Stokes et al., 2014). The following imaging parameters were employed: TEs: 4.62 ms/12.01 ms/29.04 ms/36.41 ms, TR: 1,500 ms, FOV: 32 mm^2^, matrix size: 64 × 32, in-plane spatial resolution of 0.5 × 1 mm^2^, and slice thickness: 1 mm with no inter-slice gap. The scan comprised 600 repetitions, resulting in a total acquisition time of 15 min. Additional parameters included a flip angle of 90° and slice orientation in the coronal plane with interleaved slice order. Subsequent analysis was performed on the second gradient-echo signal (TE = 12.01 ms), which is most similar to standard fMRI acquisitions.

For dMRI, data were acquired with an EPI pulse sequence with 30 directions at *b* = 670 s/mm^2^ and five non-diffusion-weighted (*b* = 0) images acquired at the beginning of the diffusion sequence. Other scan parameters include TE: 24.9 ms, TR: 11.25 s, four segments, 45 coronal slices, spatial resolution of 0.2 × 0.2 × 0.5 mm (matrix size = 150 × 150 mm, FOV 30 mm^3^), with one average. The acquisition time for DTI was 26 min 15 s.

All Bruker images were converted to NIFTI format using Bruker2niftii (https://github.com/SebastianoF/bruker2nifti) and were preprocessed using FSL (Jenkinson et al., 2012), AFNI (https://afni.nimh.nih.gov) (Cox, 1996), the Advanced Normalization Tool (ANTs; http://stnava.github.io/ANTs/) (Avants et al., 2008, 2011), and MRtrix3 (https://www.mrtrix.org/).

### 2.4 Post-mortem histology

After the study concluded, rats were transcardially perfused with Tyrode's solution followed by 4% paraformaldehyde (PFA) (Steece-Collier et al., 2019). Brains were thereafter placed in 4% PFA for an additional 72 h before cryoprotection in 30% sucrose. Brains were then sectioned using a sliding stage microtome into 40 μm serial sections and stored in cryoprotectant solution at −20°C until further use. One series was used for tyrosine hydroxylase (TH; marker of dopamine neurons) to assess the loss of dopaminergic neurons using previously described methods (Sellnow et al., 2020). Briefly, sections were washed in 1xTris buffered saline (TBS), then quenched for 30 min using 0.3% H_2_O_2_, and thereafter blocked in 10% normal goat serum. Sections were thereafter incubated with the primary antibody (1:4,000) overnight. This was followed by incubation with the secondary antibody (biotinylated horse anti-mouse IgG 1:500, BA-2001; Vector Laboratories, Burlingame, CA), followed by the ABC kit (Vectastain, Vector Laboratories, Burlingame, CA). Sections were developed using 0.5 mg/ml 3,3′-diaminobenzidine (DAB, Sigma-Aldrich, St. Louis, MO) and 0.03% H_2_O_2_. All reactions were performed at room temperature, and sections were washed in 1X TBS in between each step.

TH immunoreactive nigral neurons were enumerated from each section using an artificial intelligence platform (AIforia), as previously described (Sandoval et al., 2024) and validated (Penttinen et al., 2018). Briefly, quantification was performed on a series of sections (~7 sections), and neuronal loss was determined for each section and averaged across each animal. Because only a subset of the brain was sampled and enumerated, absolute TH+ neuron counts reported here are lower than population estimates derived from whole-brain stereological methods [typically ~21,000 neurons in intact SNc; (Penttinen et al., 2018)]. Therefore, we focused on relative comparisons between lesioned and intact hemispheres within animals, which provide a robust measure of dopaminergic cell loss in the 6-OHDA model. An insufficient lesion was thus defined as <70% loss of tyrosine hydroxylase (TH)-positive neurons in the substantia nigra pars compacta (SNc) compared to the intact hemisphere. Animals in the 6-OHDA cohort with insufficient lesioning (*n* = 3) by this criteria were removed from subsequent analyses.

### 2.5 Functional connectivity

The fMRI preprocessing involved several steps. First, the initial three time points needed to achieve steady-state magnetization were removed using fslroi (FSL) (Jenkinson et al., 2012). This was followed by despiking with 3dDespike (AFNI) to handle transient artifacts (Cox, 1996). A baseline image was created by averaging all time points using 3dTstat (AFNI), and motion correction was achieved by registering the 4D fMRI dataset to the baseline image using flirt (FSL) to ensure consistency across all 597 time points. Finally, skull stripping was performed using 3dSkullStrip (AFNI) to remove non-brain tissue.

Spatial normalization was performed in two steps using an affine transformation (ANTs). First, the GEFC image was co-registered to the SIGMA rat brain template (Barrière et al., 2019). Secondly, the baseline functional image was co-registered to the GEFC image. Subsequently, the co-registered baseline image was further transformed to the SIGMA template using the transformation matrix from the GEFC to SIGMA co-registration. The fMRI data were transformed from the baseline space to the GEFC space and then to the SIGMA template.

Finally, the normalized fMRI image was refitted to 1.5 mm and smoothed to 5 mm on voxel. To minimize partial volume effects, eroded masks for white matter, gray matter, and cerebrospinal fluid were created from the SIGMA template. Anatomical Correction (ANATICOR) was utilized to compute physiological noise regressors from local white matter signals (Jo et al., 2020). Advanced regression techniques were applied to orthogonalize the fMRI data against band-pass filters (0.01–0.1 Hz) and white matter regressors, eliminating motion and hardware-related artifacts. Functional connectivity matrices were generated using the Nilearn library in Python (https://github.com/nilearn/nilearn) using the GM mask from the SIGMA functional atlas (Table 1).

**Table 1:** The SIGMA functional atlas of rat brain.

**No**	**Hemisphere**	**Region of interest**	**No**	**Hemisphere**	**Region of interest**
**1**	Inter hemispheric	Cingulate Cortex 1	**31**	Inter hemispheric	Secondary Visual cortex
**2**	Inter hemispheric	Prelimbic Cortex	**32**	Left	Cornu Ammonis 1 (transition dorsal ventral)
**3**	Right	Primary Somatosensory	**33**	Inter hemispheric	Dorsal Lateral Periaqueductal Gray Left
**4**	Left	Primary Somatosensory	**34**	Right	Parietal Cortex (Auditory)
**5**	Inter hemispheric	Prelimbic/Infralimbic	**35**	Left	Medial geniculate nucleus
**6**	Right	Insular Cortex	**36**	Right	Dorsal Hippocampus
**7**	Inter hemispheric	Accumbens Shell	**37**	Right	Intermedial Entorhinal cortex
**8**	Right	Primary and Secondary Motor	**38**	Inter hemispheric	Retrosplenial Cortex 2
**9**	Inter hemispheric	Cingulate Cortex 2	**39**	Left	Primary and Secondary Visual Cortex
**10**	Right	Amygdala (Central, Basolateral)	**40**	Left	Dorsal Dentate Gyrus
**11**	Right	Primary Somatosensory Cortex (BE)	**41**	Inter hemispheric	Retrosplenial Cortex 3/Superior Gray
**12**	Left	Primary Somatosensory Cortex (BE)	**42**	Right	Primary and Secondary Visual Cortex
**13**	Left	Striatum (Dorsal)	**43**	Inter hemispheric	Interpeduncular nucleus
**14**	Left	Striatum (Ventral)	**44**	Right	Dorsal Dentate Gyrus/Entorhinal Cortex
**15**	Right	Striatum	**45**	Inter hemispheric	Retrosplenial Cortex 4
**16**	Left	Endo/Piriform Cortex	**46**	Right	Inferior colliculus/external cortex
**17**	Right	Insular Cortex 2	**47**	Inter hemispheric	Ventral tegmental nucleus
**18**	Inter hemispheric	Cingulate Cortex 3	**48**	Left	Cornu Ammonis 1 (Ventral)
**19**	Right	Dorsal thalamic nucleus	**49**	Left	Retrosplenial Granular Cortex Zone A/Postsubiculum
**20**	Left	Insular Cortex	**50**	Right	Retrosplenial Granular Cortex Zone A/Postsubiculum
**21**	Inter hemispheric	Hypothalamus 1	**51**	Inter hemispheric	Retrosplenial Cortex 5/Colliculus
**22**	Right	Piriform Cortex	**52**	Inter hemispheric	Pontine nuclei
**23**	Left	Dorsal Hippocampus	**53**	Inter hemispheric	Raphe (pallidum/magnun) nuclei
**24**	Right	Primary Somatosensory Cortex (Auditory)	**54**	Left	Pontine reticular/subcoeruleus
**25**	Inter hemispheric	Retrosplenial Cortex 1	**55**	Left	External colliculus (V1)
**26**	Left	Piriform Cortex	**56**	Right	Subcoeruleum/pontine reticular nucleus
**27**	Inter hemispheric	Ventral thalamic	**57**	Right	Colliculus
**28**	Inter hemispheric	Retrosplenial Granular Cortex c	**58**	Right	RSD/RSGa
**29**	Left	Parietal Cortex (Auditory)	**59**	Right	Raphe/Median(paramedian) pontine reticular nu
**30**	Inter hemispheric	Hypothalamus 2	

### 2.6 Structural connectivity

For dMRI preprocessing, denoising was performed using *dwidenoise* (MRtrix3) (Veraart et al., 2016). This was followed by distortion correction using *tortoise* (https://tortoise.nibib.nih.gov/) and eddy current correction using *eddy* (FSL) (Andersson and Sotiropoulos, 2016). Brain extraction from the averaged b0 images was performed with *dwi2mask* (MRtrix3). Subsequently, all preprocessed dMRI images were resampled to a voxel size of 1.5 mm for normalization to the standard space. Tractography was performed using the *tckgen* tool (MRtrix3) with a deterministic algorithm (Basser et al., 2000). The parameters for tractography included nthreads 8, step size 0.8, minimum length 10, maximum length 220, and 2 million seeds. The seed image was a mask encompassing the white matter/gray matter border.

Structural connectivity matrices were generated with *tck2connectome* (MRtrix3) using the SIGMA Functional Brain Atlas. This template was co-registered to each diffusion native space using the ANTs symmetric image normalization method (SyN). Finally, each connectome was filtered (to remove incomplete tracts) using the *connectome2tck* tool (MRtrix3). As for functional connectivity, structural connectivity matrices were derived using the SIGMA Functional Brain Atlas (Table 1).

### 2.7 Free-water DTI maps

For the computation of fw-corrected DTI metrics, a custom MATLAB (MATLAB R2022b) script for single-shell fw-DTI (Pasternak et al., 2009) was used after dMRI preprocessing (described above). These metrics included fractional anisotropy (fw-FA), axial and radial diffusivities (fw-AxD and fw-RD, respectively), and the fw index (f). fw-FA reflects the directionality of water diffusion associated with white matter fibers, while fw-AxD captures diffusion along the primary fiber direction, and fw-RD assesses diffusion perpendicular to this direction. The f metric quantifies the amount of free water in the extracellular space. fw-FA, fw-AxD, and fw-RD are corrected metrics that account for the presence of free water, providing a more accurate representation of tissue microstructure by reducing the influence of extracellular water on diffusion measurements (Pasternak et al., 2009).

### 2.8 Statistical analysis

One-sample two-tailed *t-*tests were conducted respectively on the functional and structural connectivity matrices from the 6-OHDA and sham groups across all 59 regions of interest (ROIs, Table 1). A significance matrix was created using a *p* < 0.01 threshold to identify significant connections. This significance matrix was then applied to filter the connectivity matrices, highlighting only the significant connections.

For both functional and structural connectivity analyses, a two-sample *t-*test was implemented to compare the two groups using a linear model with post-surgery time as a covariate in R and R studio script (R version 4.3.1). Resulting *p-*values were corrected using the Benjamini and Yekutieli (BY) False Discovery Rate (FDR) procedure (Benjamini and Yekutieli, 2005), with a threshold of FDR < 0.05 to identify significant connections. Additionally, effect sizes were calculated using Hedges' g value (g). For statistically significant connections, brain connectivity was visualized using BrainNetViewer Version 1.7 (Xia et al., 2013).

Significant structural connectivity connections were analyzed using free water-corrected DTI metrics, and voxel-based analysis was performed within the significant tracts. The Threshold-Free Cluster Enhancement (TFCE) method was utilized for this analysis to ensure robust clustering while avoiding arbitrary thresholding and addressing multiple comparisons.

## 3 Results

### 3.1 Behavioral test and histological data

**Figure 1:**
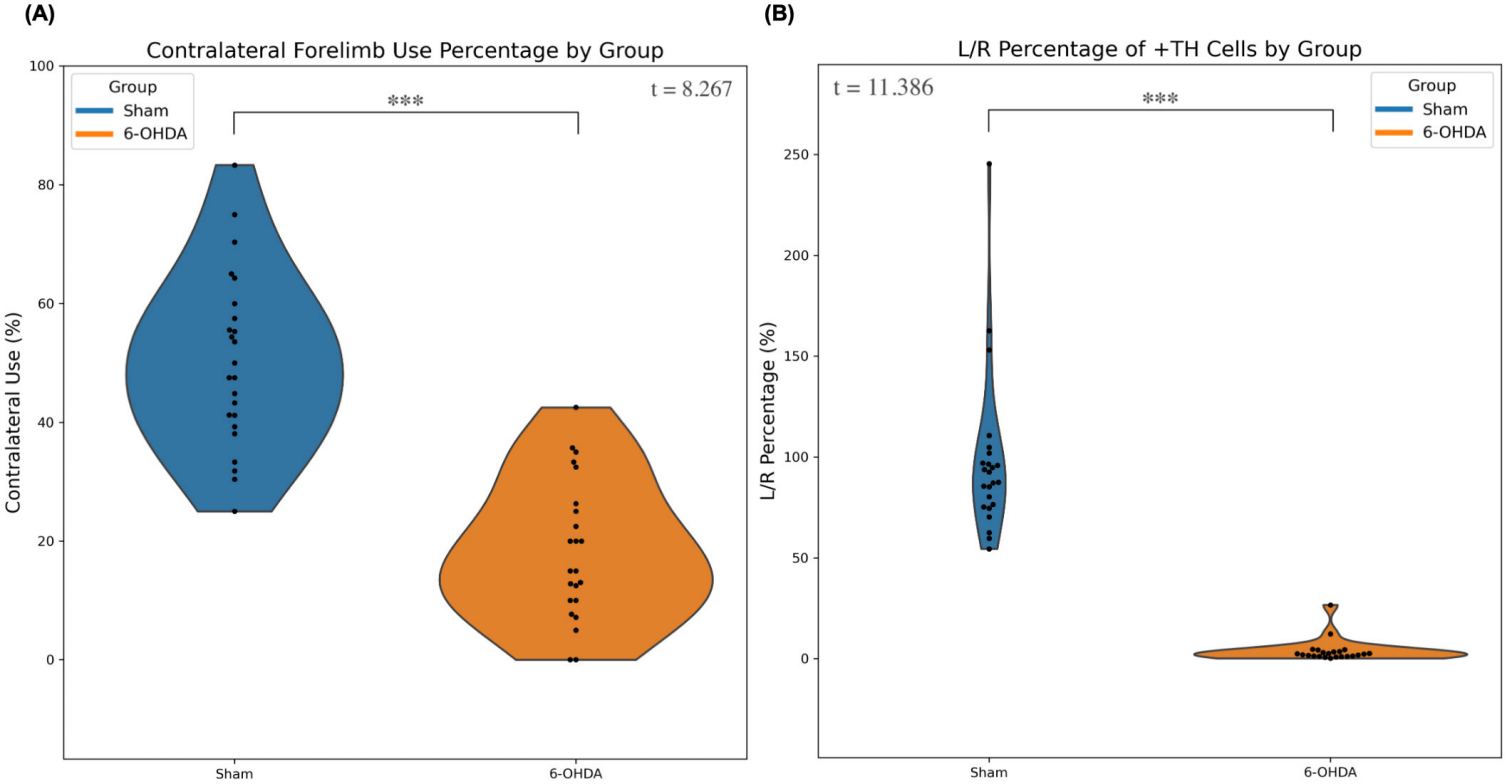
Motor impairment and TH+ cell density in 6-OHDA and sham groups. **(A)** Contralateral forelimb use was significantly reduced in the 6-OHDA group compared to the sham group (*p* < 0.001, quantified in Table 2). **(B)** L/R ratio showed a marked reduction in the 6-OHDA group vs. the sham group (*p* < 0.001, quantified in Table 2). ^***^Indicates *p* < 0.001.

In the 6-OHDA model, a significant impairment in the contralateral paw (mean ± SD: 18.3% ± 11.8%) was observed vs. sham (mean ± SD: 50.3% ± 14.6%) (*t* = 8.267, *p* < 0.001), as shown in Figure 1A. Post-mortem histology revealed significant TH+ cell loss in the 6-OHDA group compared to the sham group (Figure 1B and Table 2). In the sham group, the mean number of TH+ cells in the left hemisphere was 821.4 ± 310.3, whereas the 6-OHDA group exhibited a significant depletion, with only 24.3 ± 36.4 cells (*t* = 12.494, *p* < 0.001). In contrast, TH+ cell counts in the right hemisphere were comparable between groups, with the 6-OHDA group showing 731.4 ± 300.3 cells and the sham group 894.3 ± 332.0 cells (*p* = 0.084), indicating no significant difference (Table 2). Accordingly, the ipsilateral/contralateral TH+ cell ratio was drastically reduced in the 6-OHDA group (3.7% ± 5.6%) compared to the sham group (97.8% ± 40.1%), with a highly significant difference (*p* < 0.001), confirming the severe asymmetry induced by the 6-OHDA lesion (Table 2).

**Table 2:** Comparison of behavioral impairment and TH+ cell loss in the 6-OHDA and sham group.

**Group**	**Behavioral test (% ±SD)**	**Left TH+ cell count (cell count ±SD)**	**Right TH+ cell count (cell count ±SD)**	**Left/right ratio (% ±SD)**
6-OHDA	18.3% ± 11.8%	24.3 ± 36.4	731.4 ± 300.3	3.7% ± 5.6%
Sham	50.3% ± 14.6%	821.4 ± 310.3	894.3 ± 332.0	97.8% ± 40.1%
*p*-value	<0.001	<0.001	0.084	<0.001

### 3.2 Functional connectivity

**Figure 2:**
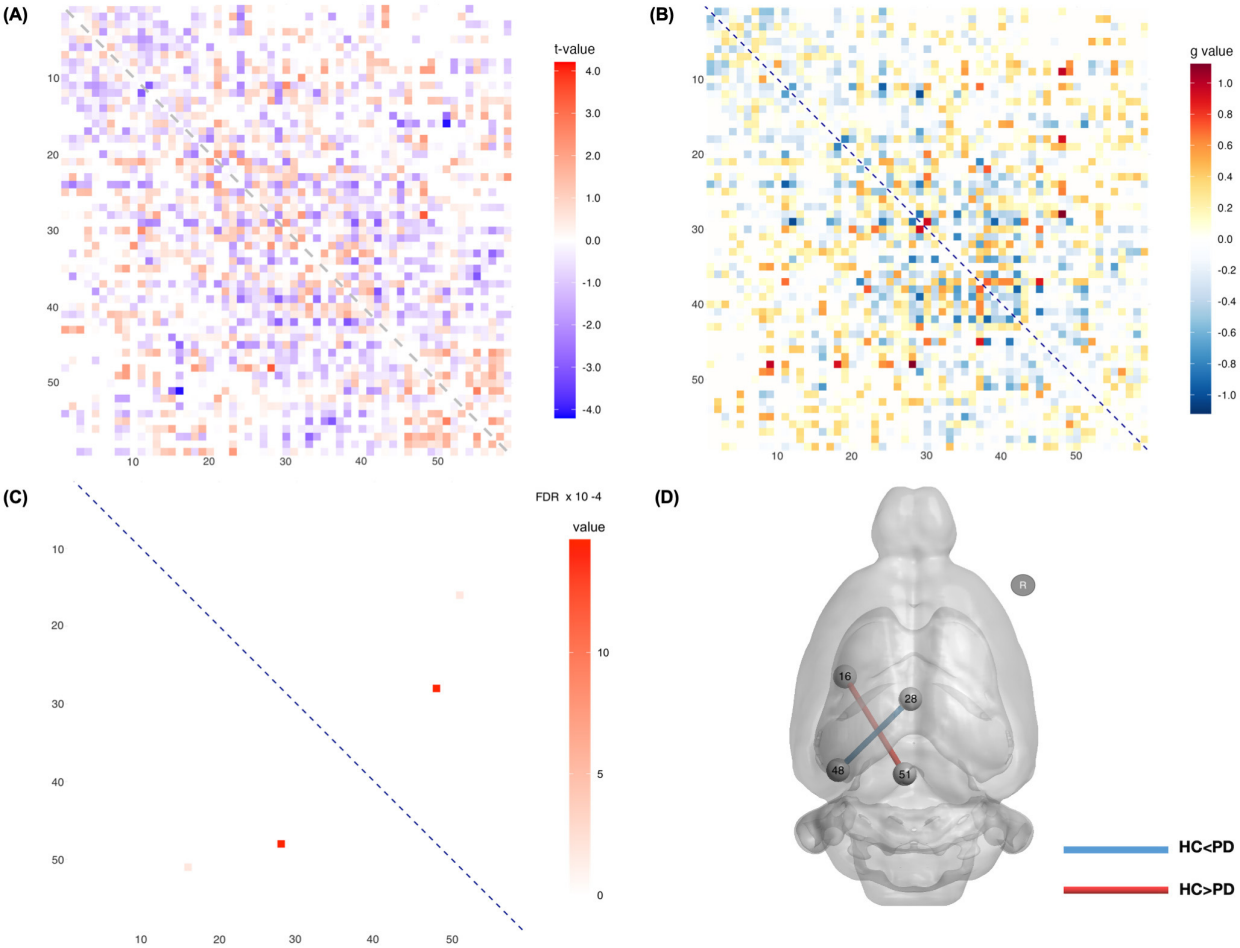
Functional connectivity between the sham and 6-OHDA groups. The left hemisphere (L) is defined as the lesion side. **(A)** Matrix displaying *t-*values across connections, where blue denotes reduced connectivity and red denotes enhanced connectivity in 6-OHDA rats. **(B)** Matrix illustrating corresponding effect size across connections. **(C)** Matrix displaying connections with significant FDR-corrected *p-*values (*p* < 0.05). **(D)** Visualization of the spatial locations of these significant connectivity differences. Specifically, compared to the sham group, a significant increase in functional connectivity was found between the ipsilateral left cornu ammonis and retrosplenial granular cortex in 6-OHDA rats, while a significant decrease was observed between the ipsilateral left retrosplenial cortex and endopiriform cortex. The statistical details for these differences are presented in Table 3. R, right hemisphere.

For FC analysis, three rats (6-OHDA: *n* = 1; sham: *n* = 2) were excluded due to incomplete data; thus, the analysis included 46 rats (6-OHDA: *n* = 23; sham: *n* = 23). Figure 2A displays a map of the *t-*values representing the connectivity strength between each ROI, with blue indicating reduced and red indicating increased connectivity in the 6-OHDA rats compared to sham rats. Figure 2B shows the effect size (g) for all connections. Significant differences in functional connectivity were observed in several regions of the lesioned hemisphere, as highlighted in Table 3. The FDR corrected *p-*values for the significant connections are shown in Figure 2C. Specifically, a significant decrease in FC for the 6-OHDA rats was found between the ipsilateral left retrosplenial cortex and endopiriform cortex (*t* = −4.097, *g* = −0.714, FDR corrected *p* = 0.002). A significant increase in functional connectivity was found between the ipsilateral left cornu ammonis and retrosplenial granular cortex (*t* = 3.400, *g* = 1.107, FDR corrected *p* < 0.001). Figure 2D provides a visualization of the spatial locations of these significant connectivity differences. Blue lines indicate stronger connectivity in the 6-OHDA group, whereas red lines indicate stronger connectivity in the sham group.

**Table 3:** Statistical differences in functional connectivity between the two groups were observed in two connections (FDR < 0.05).

**Node 1**			**Node 2**			**FDR**	***t***	***g***	**Comparison (HC vs. PD)**
48	Left	Cornu Ammonis 1 (Ventral)	28	Inter hemispheric	Retrosplenial Granular Cortex c	0.002	3.400	1.107	HC < PD
51	Inter hemispheric	Retrosplenial Cortex 5/Colliculus	16	Left	Endo/Piriform Cortex	<0.001	−4.097	−0.714	HC> PD

### 3.3 Structural connectivity

**Figure 3:**
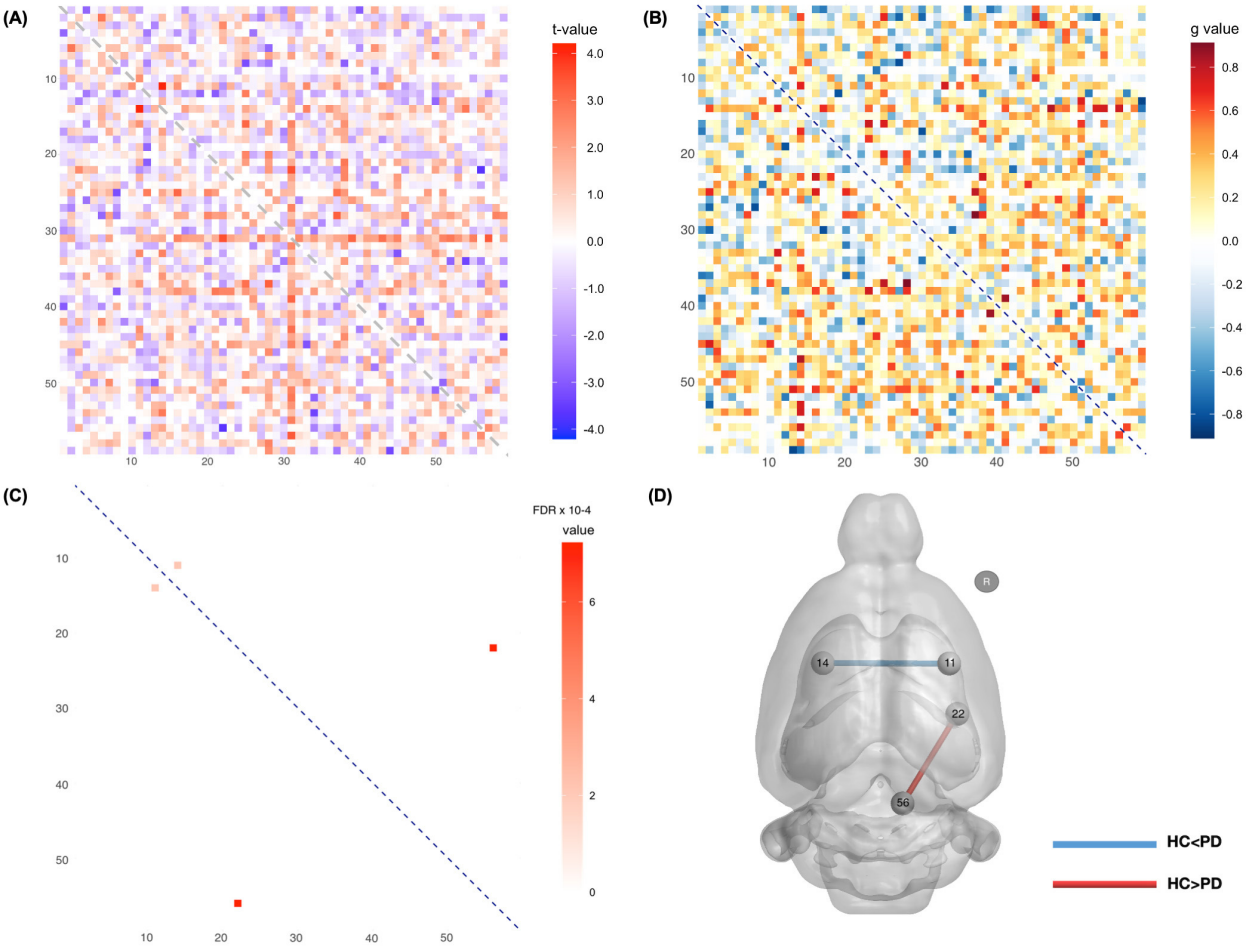
Structural connectivity between the sham and 6-OHDA groups. **(A)** Matrix displaying *t-*values across connections, where blue denotes reduced connectivity and red denotes enhanced connectivity in 6-OHDA rats. **(B)** Matrix illustrating the variability in effect size across connections. **(C)** Matrix displaying connections with significant FDR-corrected *p-*values (*p* < 0.001). **(D)** Provides a visualization of the spatial locations of these significant connectivity differences. Specifically, compared to the sham rats, a significant increase in SC in the 6-OHDA rats was found between the left ventral striatum and the right primary somatosensory cortex, while a significant decrease in SC was found between the right subcoeruleum/pontine reticular nucleus and the right piriform cortex. The statistical details for these differences are presented in Table 4. R, right hemisphere.

For SC analysis, five rats (6-OHDA: *n* = 4; sham: *n* = 1) were excluded due to incomplete data; thus, the analysis included 44 rats (6-OHDA: *n* = 20; sham: *n* = 24). Figure 3A displays a map of the *t-*values representing the connectivity strength between ROIs, with blue indicating reduced and red indicating increased connectivity in the 6-OHDA rats, compared to the sham rats. Figure 3B shows the effect size for all connections. Significant differences in structural connectivity were observed in several regions, as shown in Table 4, and the FDR corrected *p-*values for the significant connections are shown in Figure 3C. Compared to the sham rats, a significant increase of SC in the 6-OHDA rats was found between the left ventral striatum and the right primary somatosensory cortex (*t* = 4.068, *g* = 0.701, FDR corrected *p* < 0.001). In contrast, a significant decrease of SC was found between the right subcoeruleum/pontine reticular nucleus and the right piriform cortex (*t* = −3.658, *g* = −0.915, FDR corrected *p* < 0.001). Figure 3D provides a visualization of the spatial locations of these significant connections. Blue lines indicate stronger connectivity in the 6-OHDA group, whereas red lines indicate stronger connectivity in the sham group.

**Table 4:** Statistical differences in structural connectivity between the two groups were observed in two connections (FDR < 0.001).

**Node 1**			**Node 2**			**FDR**	***t***	***g***	**Comparison (HC vs.PD)**
14	Left	Striatum (Ventral)	11	Right	Primary Somatosensory Cortex (BE)	<0.001	4.068	0.701	HC < PD
56	Right	Subcoeruleum/pontine reticular nucleus	22	Right	Piriform Cortex	<0.001	−3.656	−0.915	HC> PD

### 3.4 Free-water DTI

**Figure 4:**
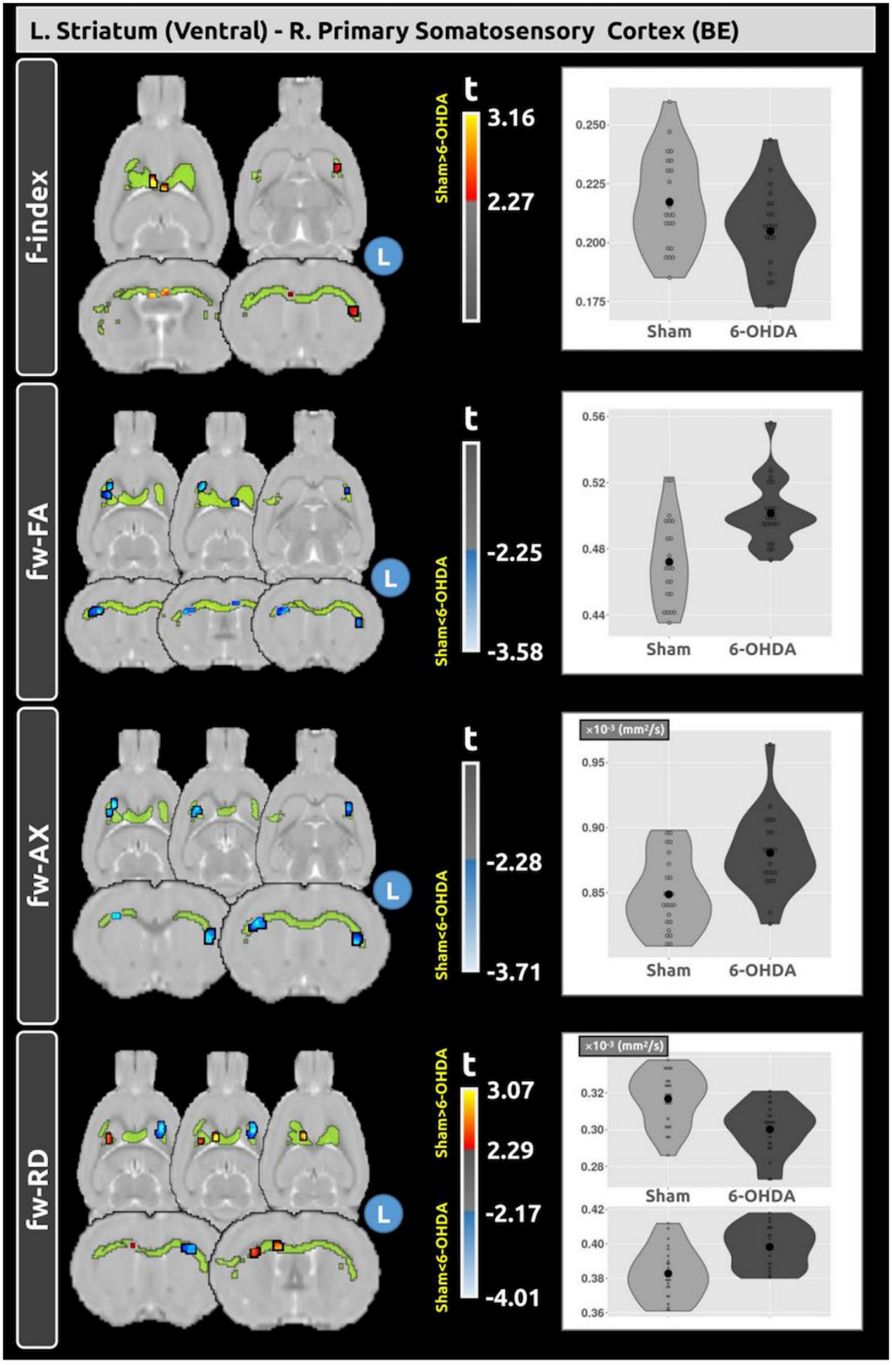
Voxel-based analysis reveals differences in fw-DTI metrics within the tract connecting the left ventral striatum and the right primary somatosensory cortex between the sham and 6-OHDA groups. A significant increase in structural connectivity was observed in the 6-OHDA group compared to the sham group (*t* = 4.068; FDR < 0.001). Within this tract, distinct clusters show lower f-index and increased fw-FA and fw-AxD in the 6-OHDA group. Plots depict the average values of the fw diffusion metrics within these significant clusters for both groups.

Voxel-based analysis identified significant differences across single-shell fw-DTI metrics in the tracts connecting nodes with significant changes in structural connectivity. A significant increase in structural connectivity was found between the left ventral striatum and the right primary somatosensory cortex. Voxel-based analysis was conducted for the fw-DTI metrics within this tract, revealing clusters of significant differences between the sham and 6-OHDA groups, as shown in Figure 4. The f-index showed lower values in the 6-OHDA group compared to the sham group. For fw-FA, the 6-OHDA group exhibited higher values compared to sham rats. The fw-AxD metric also showed higher values in the 6-OHDA group, while fw-RD showed clusters with both increased and decreased values.

**Figure 5:**
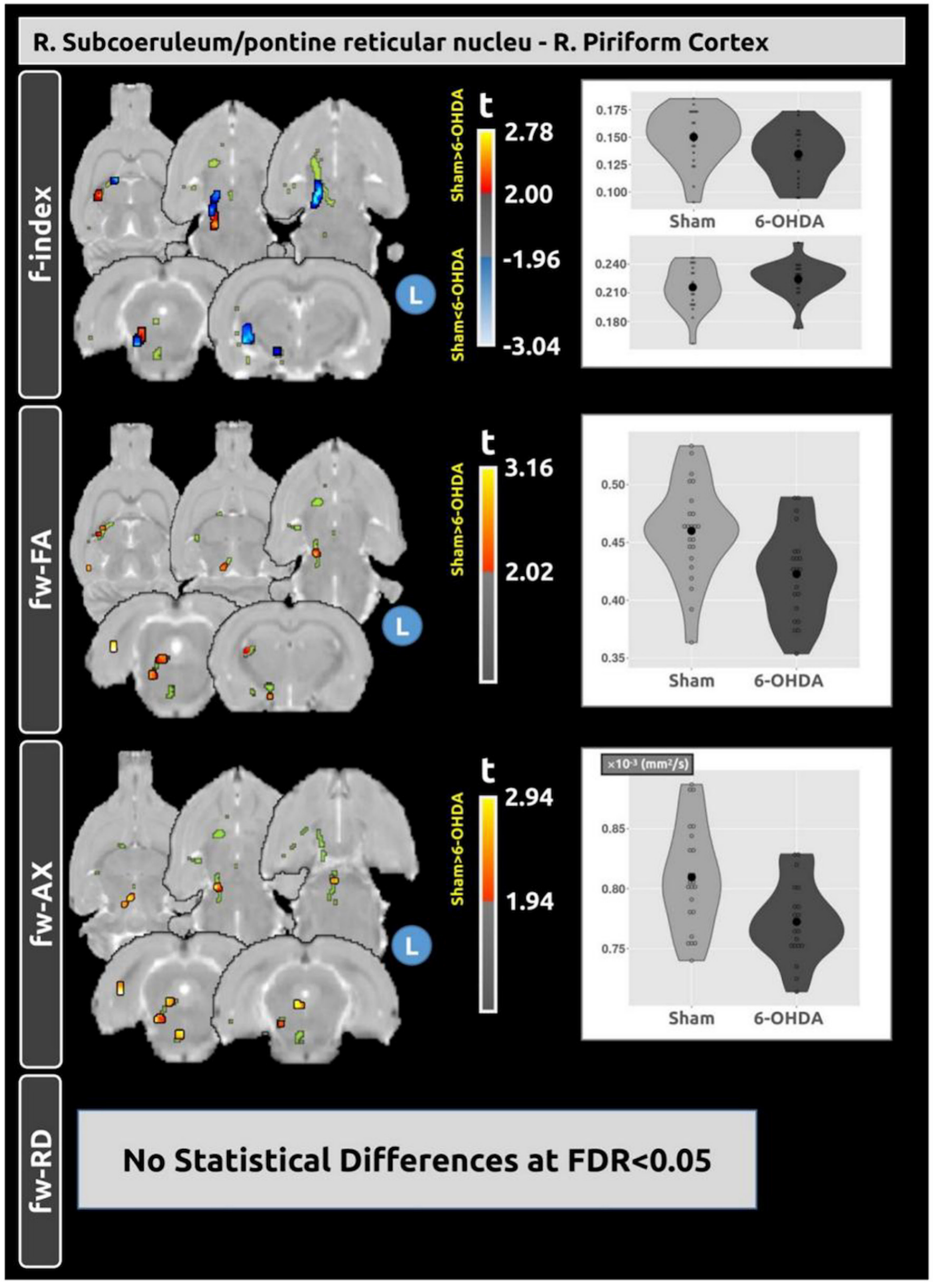
Voxel-based analysis reveals differences in fw-DTI metrics within the tract connecting the right subcoeruleus/pontine reticular nucleus and the right piriform cortex between the sham and 6-OHDA groups. A significant decrease in structural connectivity was observed in the 6-OHDA group compared with the sham group (*t* = −3.656; FDR < 0.001). Within this tract, distinct clusters of differences in f-index, fw-FA, and fw-AxD were observed between the groups. No statistical differences were found for fw-RD. Plots depict the average values of the fw-DTI metrics within these significant clusters for both groups.

A significant decrease in structural connectivity was found between the right subcoeruleus/pontine reticular nucleus and the right piriform cortex. Voxel-based analysis within this tract revealed significant clusters of differences between the sham and 6-OHDA groups, as shown in Figure 5. The f-index showed both higher and lower average values in the 6-OHDA group compared to the sham group. The 6-OHDA group exhibited lower fw-FA and fw-AxD values in the 6-OHDA group compared to the sham group. For fw-RD, no significant differences were found between the groups in this tract.

## 4 Discussion

In this study, we found significant differences in both FC and SC between 6-OHDA lesioned rats and sham-operated controls, reflecting the impact of PD-related pathology on the integrity of the brain's networks in rats. While SC reflects the physical pathways through which brain signals travel, FC reveals how these pathways are functionally utilized during brain activity. Combined analysis of FC and SC may be useful to identify early stages of network disruption, understand the brain's compensatory mechanisms, and monitor how these changes evolve over time (Filippi et al., 2020).

The 6-OHDA lesion model is a widely used and well-characterized model that replicates many clinical motor and non-motor features of PD, with the flexibility to adjust disease severity based on injection sites and toxin dosage (Kirik et al., 1998). The injection of 6-OHDA into the SNc causes rapid and selective degeneration of dopaminergic neurons within SNc (Perlbarg et al., 2018), while injection into the our targeted area of MFB results in almost total destruction of dopaminergic neurons of the SNc projecting to striatum, as well as the ventral tegmental area (VTA) heading to the nucleus accumbens, eventually causing a denervation-induced postsynaptic sensitivity of dopaminergic receptors (Sellnow et al., 2020). The SN+MFB targeting results in widespread damage across the entire dopaminergic system, thus establishing a comprehensive late stage PD model. The associated behavioral manifestations closely resemble motor dysfunction in clinical PD, making it a valuable model for studying the role of dopaminergic loss in PD (Eskow Jaunarajs et al., 2010, 2012; Carvalho et al., 2013). In the present study, the validity of this model was confirmed through both behavioral testing and histological analysis.

In previous studies, the 6-OHDA rat model has offered valuable insights into the neuroimaging patterns associated with PD. For instance, Perlbarg and colleagues demonstrated significant FC and SC alterations, especially in brain regions and networks associated with motor control, sensory processing, and cognitive functions (Perlbarg et al., 2018). Increased FC has been observed in 6-OHDA-lesioned rats, with Westphal et al. reporting bilateral thalamic FC increases and Monnot et al. noting heightened connectivity between lesion sites and the sensorimotor cortex (Monnot et al., 2017; Westphal et al., 2017). These findings underscore the robustness of the 6-OHDA model in capturing neural network alterations. With regard to structural analysis, reduced structural biomarkers have been observed in lesioned rats, correlating with motor dysfunction (Zhang et al., 2017). Moshchin et al. using correlational tractography identified negatively correlated tracts between the striatum and substantia nigra pars compacta in 6-OHDA rats (Moshchin et al., 2023).

Building on these previous findings, our study further analyzes the FC and SC changes in the 6-OHDA-lesioned rat model to provide insights into PD-related network disruptions. In our study, both increased and decreased FC involving the retrosplenial cortex (RSC) were observed, highlighting the complex role this region plays in degenerative and compensatory processes. The RSC, including its granular subregion RSGC, plays a critical role in processing and integrating spatial and contextual information. The RSC is important in episodic memory and changes to the connectivity in this structure has been implicated to drive episodic memory impairment in PD (Brennan, 2021; Crowley et al., 2021). Previous research has shown increased FC between the RSC and the posterior cingulate cortex (PCC) in PD patients, particularly those at Hoehn and Yahr stage II, suggesting enhanced integration within the DMN (Luo et al., 2015). Interestingly, our study observed increased connectivity between the hippocampus—important in the DMN—and the RSC. These regions are known to be anatomically connected, with tracer-based studies confirming projections between the retrosplenial cortex and hippocampal subfields such as CA1 (Sugar et al., 2011). While our study did not identify specific structural changes related to RSC, it has been reported that SC within the RSC partially reflects its FC with different subregions exhibiting distinct connectivity patterns (Li et al., 2018). Notably, similar increases in hippocampus-RSC connectivity have been documented in other neurodegenerative diseases, such as Alzheimer's Disease (AD), highlighting a potential common pathway for network reorganization (Ziontz et al., 2021). In PD, while the DMN shows widespread hypoconnectivity in cognitive regions such as prefrontal cortex, increased connectivity is often observed in motor-related networks, potentially as a compensatory response (Tessitore et al., 2012; Onu et al., 2015; Chen et al., 2022). This compensation for functional deficits may help maintain somatosensory performance despite dopaminergic loss, with the increased HC-RSC connectivity suggesting broader DMN involvement in compensatory processes to mitigate clinical decline in PD.

On the other hand, decreased connectivity between the endopiriform cortex and RSC was observed in our study. The endopiriform cortex is involved in integrating sensory information, particularly olfactory inputs; changes in this region may reflect a broader disruption in the brain's ability to integrate sensory and contextual information, likely due to the cascading effects of dopaminergic depletion on connected networks (Sugai et al., 2012). Similarly, decreased SC between the ipsilateral subcoeruleum/pontine reticular nucleus (areas showing degeneration or pathology in PD) and piriform cortex in 6-OHDA rats further highlights the role of dopaminergic loss in impairing both functional and structural sensory pathways. The piriform cortex, critical for olfactory processing, is especially relevant to early PD symptoms like loss of smell (Sugai et al., 2012). These parallel declines in FC and SC across sensory-related regions suggest that the effects of degeneration propagate through interconnected networks, resulting in widespread impairments in sensory integration, particularly of olfactory signals. Similarly, reduced FC in the somatosensory cortex and ventral posterior medial nucleus has been observed in 6-OHDA rats (Westphal et al., 2017), aligning with human studies showing sensory-motor cortex hypoconnectivity in PD and further highlighting the impact of dopaminergic neuron loss on sensory networks (Wang et al., 2024).

Previous studies have reported widespread reductions in SC in PD patients (Tinaz et al., 2017). Though our findings were more localized, decreased structural connectivity between the piriform cortex and subcoeruleum may reflect broader disruptions linked to the locus coeruleus (LC), a region affected by noradrenergic dysfunction in PD and reported in previous studies to be impacted by 6-OHDA lesion (Tredici and Braak, 2013; Shin et al., 2014). As the LC supports motor and sensory integration through extensive projections, its degeneration may drive early-stage network disruptions, signaling the onset of neurodegenerative changes preceding widespread sensory and cognitive impairments. Indeed, the 6-OHDA lesion procedure used in the current study did not employ the noradrenergic transport blocker desipramine to protect the LC, further uncovering its potential network influences (Barnum et al., 2012; Shin et al., 2014).

Increased SC was observed between the ipsilateral ventral striatum and the contralateral primary somatosensory cortex in PD rats, which was confirmed by a medium effect size. This structural connection was supported by tract-tracing studies demonstrating projections from the somatosensory cortex to the ventral striatum (Hunnicutt et al., 2016). Previous studies have demonstrated that dopaminergic denervation results in significant alterations in white matter tracts within the striatum both in the 6-OHDA rat model and humans, as evidenced by increased AxD, MD, and f-index (Perlbarg et al., 2018; López-Aguirre et al., 2023). The brain may compensate for these neurodegenerative changes by strengthening SC within the somatosensory cortex, thereby supporting the integration of sensory input with motor actions, which rely heavily on the interaction between these regions. Moreover, it has been suggested that strong SC between two areas increases the likelihood and strength of corresponding functional connectivity (Díaz-Parra et al., 2017). However, there is concern that increased SC observed in cross-sectional tractography may also reflect non-compensatory changes, including edema, gliosis, or fiber orientation alterations (Maier-Hein et al., 2017). Nonetheless, the SC increase observed in our study may still provide a clue to potential compensatory attempt by the brain to preserve functionality, further supported by evidence of increased FC between the striatum and somatosensory cortex in early-onset human PD (Hou et al., 2016).

It is important to note that not all observed connections in this study are currently supported by direct tract-tracing evidence. While key tracts such as RSC–hippocampus and somatosensory–ventral striatum are anatomically validated, other tracts identified through tractography may reflect polysynaptic integration or compensatory rewiring. As such, these findings warrant further anatomical validation using tracer-based methods.

To deepen our understanding of these SC changes, voxel-based fw-DTI analysis was employed to further investigate metrics within the two distinct tracts that exhibited SC changes. In the tract connecting the right subcoeruleus/pontine reticular nucleus and the right piriform cortex, the observed SC decrease aligns with expected patterns of neurodegeneration in 6-OHDA (Perlbarg et al., 2018), where compromised axonal integrity (reflected by lower fw-FA and fw-AxD) may contribute to connectivity reductions. Interestingly, the lack of significant changes in fw-RD suggests relatively preserved myelin integrity. This aligns with the broader understanding that free-water changes in PD reflect disruptions in gray matter and iron accumulation, offering additional insights into PD-related structural changes (Ofori et al., 2015b), though such interpretations should be made cautiously due to the limitations of single-shell data. In the tract connecting the ipsilateral ventral striatum and contralateral primary somatosensory cortex, the increased SC observed in the 6-OHDA group was accompanied by a lower f-index, indicating reduced extracellular free water, as well as higher fw-FA and fw-AxD values. These findings may reflect a potential compensatory mechanism, where the brain adapts to neurodegenerative changes to preserve motor and sensory functions. Supporting this concept, lower f-index values have been linked with slower progression of bradykinesia over 1 year (Ofori et al., 2015b). Overall, decreased SC was associated with variable f-index values (both higher and lower) and lower fw-FA and fw-AxD, while increased SC corresponded to lower f-index and higher fw-FA and fw-AxD. These findings align with previous studies highlighting the role of free-water metrics within the substantia nigra as markers of disease progression and compensatory adaptations in Parkinson's disease (Ofori et al., 2015a,b).

Ortiz et al. (2015) demonstrated that combining FC and SC patterns enhances the accuracy of neurodegenerative disease diagnosis, and similar approaches have been applied to predict cognitive decline in PD (Chen et al., 2015; Ortiz et al., 2015). Our findings support this integration by highlighting the underlying mechanisms within the significant tracts, thereby strengthening the reliability of these biomarkers. This underscores the value of integrating FW analysis with SC and FC, offering a comprehensive understanding of how PD affects brain connectivity. Although validation with multi-shell data is needed, our findings are supported by convergence across multiple diffusion metrics and tract-level connectivity alterations, suggesting biological relevance despite methodological constraints.

There are several limitations in this study. First, the dMRI acquisition included only a single b-value, and the lack of multiple b-values makes it difficult to precisely separate different diffusion compartments in the bi-exponential model fitting (Pasternak et al., 2009). Moreover, free water estimates from single shell data are sensitive to initial conditions, which may not hold true in complex tissue environments (Bergmann et al., 2020). Future studies could leverage multi-shell dMRI data to better estimate both the free water volume and tissue-specific diffusion parameters. Another limitation of our study is the use of fMRI network analysis in rats, particularly under the influence of isoflurane anesthesia. Isoflurane is known to suppress neural function, particularly in cortical and subcortical regions, potentially impacting the accuracy of network dynamics observed in the DMN and sensory-motor systems (Li and Zhang, 2018). To overcome this challenge, a standardized low-dose of isoflurane was used during fMRI, and ANATICOR was used to minimize confounding effects. However, the use of anesthesia may still attenuate FC strength and limits the direct comparability of these findings to those obtained from awake human PD studies. This limitation should be considered when interpreting FC results, and future studies incorporating awake imaging paradigms may enhance translational validity. Additionally, applying human fMRI analysis methods to rat brains introduces challenges due to species differences in brain structure and connectivity patterns, complicating direct comparisons (Xu et al., 2022). This study also included only male rats, which limits the generalizability of our findings to both sexes. Given sex differences known in PD, including greater disease burden in females and differences in dopaminergic and hormonal pathways (Picillo et al., 2017), future studies should include female animals to assess sex as a biological variable. Finally, only two significant differences were detected in both SC and FC after FDR correction; this limited number of significant findings may reflect modest effect sizes, underlying biological variability, or the conservative corrections thresholds. Future studies with larger sample sizes and improved signal-to-noise ratios may help to clarify additional network alterations. Despite these limitations, fMRI remains a crucial tool for investigating PD-related network disruptions. Future research should focus on refining these techniques, including the use of awake imaging, to mitigate anesthesia-related biases and improve translatability to human studies.

## 5 Conclusion

In conclusion, our study demonstrated key changes in both functional and structural connectivity in the 6-OHDA rat model of PD; changes that are analogous to that seen in human disease. Functionally, decreased connectivity between the retrosplenial and endopiriform cortices suggests disrupted sensory processing, while increased connectivity between the hippocampus and retrosplenial cortex indicates possible compensatory mechanisms. Structurally, we observed reduced connectivity between the subcoeruleum and piriform cortex, which may reflect axonal degeneration, and increased connectivity between the ventral striatum and primary somatosensory cortex, which likely reflects compensatory changes to support motor-sensory integration. FW-DTI analysis further revealed changes in the white matter tracts connecting these regions, supporting these findings and highlighting adaptive responses to neurodegeneration in PD. These findings offer important insights into PD progression, and further research is necessary to determine their potential as biomarkers for monitoring disease progression or evaluating therapeutic interventions.

## Data Availability

Data and code may be made available upon the construction of a formal data sharing agreement.
